# Prof. P. H. Austen, M. D., D. D. S.

**Published:** 1874-02

**Authors:** 


*Prof. P. H. Austen, M. D., D. D. S.*—The many friends of Prof. Austen will be pleased to learn that he has so far recovered from his late severe indisposition, that he has for some time past been delivering lectures in the Baltimore College of Dental Surgery, and expects to continue doing so throughout the session.

